# Is the Growth of the Fetus of a Non-Smoking Mother Influenced by the Smoking of Either Grandmother while Pregnant?

**DOI:** 10.1371/journal.pone.0086781

**Published:** 2014-02-04

**Authors:** Laura L. Miller, Marcus Pembrey, George Davey Smith, Kate Northstone, Jean Golding

**Affiliations:** 1 School of Social and Community Medicine, University of Bristol, Bristol, United Kingdom; 2 Institute of Child Health, University College London, London, United Kingdom; National Taiwan University, Taiwan

## Abstract

**Background:**

There are animal data that indicate that prenatal environmental exposures have sex-specific effects on subsequent generations. In humans, an increase in birthweight has been reported if the maternal grandmother had smoked in the pregnancy giving rise to the mother. Here we assess whether prenatal exposure of either parent to cigarette smoke has a sex-specific effect on the grandchild's birth measurements.

**Methods:**

Information from 12707 maternal and 9677 paternal grandmothers of children in the Avon Longitudinal Study of Parents and Children (ALSPAC) concerned whether they had smoked while expecting the study parent. Study children were weighed and measured at birth. Analyses to test effects of grandmaternal prenatal smoking used multiple regression allowing for several potential confounders; analyses were restricted to births to non-smoking study mothers.

**Findings:**

After adjustment, the average birthweight, birth length and BMI measurements of the grandsons (but not granddaughters) were greater if the maternal grandmother smoked prenatally: birthweight  = +61 [95% CI +30, +92] g; birth length  = +0·19 [95% CI +0·02, +0·35] cm; BMI  = +1·6 [95% CI +0·6, +2·6] g/m^2^. Similar effects were seen in births to primiparae and multiparae. Additional allowance for maternal birthweight resulted in an average increase in boys to +100 g [95% CI +61, +140] g. There were no fetal growth differences if the paternal grandmother had smoked prenatally.

**Conclusions:**

The evidence from this study suggests that when the mother does not smoke in pregnancy the maternal grandmother's smoking habit in pregnancy has a positive association with her grandson's fetal growth.

## Introduction

This study of transgenerational effects was instigated following studies from Sweden based on samples of individuals born in the town of Överkalix in specified years. Their longevity and other health outcomes were linked to detailed historical records of harvests experienced by their ancestors. Analyses using three independent cohorts showed that the paternal grandfathers' plentiful food supply in mid-childhood was associated with a four-fold increased chance of diabetes on the grandchild's death certificate [1]. Subsequently sex-specific analyses of the data showed that the mortality rates of the men born in the target years were linked to the quality of the paternal grandfather's food supply in mid-childhood, whereas the mortality rate of the women studied was associated solely with their paternal grandmother's food supply [2], [3]. The exposure sensitive periods were in the paternal grandparents' mid-childhood and in the fetal/infant period for paternal grandmothers. These Överkalix results strongly suggest that a trans-generational response occurred to influences during the grandmothers' own fetal/infant period but that this only affected the female grandchildren born to her sons.

Although increasing interest is being shown in transgenerational effects on growth and development, few studies have looked at effects on fetal growth. Adult mortality is known to be associated with birthweight differentially by cause of death [4]. It is logical therefore to investigate whether fetal growth is a marker for transgenerational environmental influences and, if so, whether these differ by the gender of the offspring (given the sex-specific effects found in the Överkalix study).

In this paper we use maternal prenatal cigarette smoking as our exemplar since it has strong effects on fetal growth. However it is not clear whether there is an effect on the subsequent generations. Only three studies to our knowledge have looked at possible transgenerational effects in regard to prenatal smoking. Misra and colleagues [5] analysed data on maternal and grandmaternal smoking from the Baltimore branch of the US Collaborative Perinatal Project. We have calculated from data presented in their paper that among their 989 study subjects there was a 244 g increase in offspring of non-smoking mothers if the mothers' own mother had smoked prenatally. This was after adjustment for infant gender and maternal birthweight, maternal race, hospitalisation in childhood, height, age, education and poverty level, grandmaternal height, BMI at the time of maternal birth, grandmaternal education, poverty and history of sexually transmitted diseases; an interaction between grandmaternal smoking and parity of the pregnancy of the grandchild was reported. Similarly, in a study following up births to non-smoking women participating in the British 1958 birth cohort, after adjustment for gestation, maternal birthweight, maternal height and BMI, there was a mean increase of +45 [95% CI +10, +80] g if the mother had been exposed to her mother smoking *in utero*, but no allowance was made for parity and the measurement of birthweight tended to be rounded to the nearest 4 oz. before conversion to gm [6]. Analyses of the Michigan Bone Health and Metabolism Study [7] showed a 154 g increase in birthweight of offspring of mothers who had themselves been exposed prenatally, after adjustment for a variety of confounders including maternal smoking, parity, maternal birthweight and weight gain in pregnancy. None of these analyses assessed whether there were different effects according to gender of the offspring. Nor have there been studies, to our knowledge, assessing whether the father's prenatal exposure to his mother smoking has any effect on the growth of the fetus of his non-smoking partner.

The aim of the present study is therefore to determine whether prenatal smoke exposure of either of the study child's parents influences the growth of the fetus – and to determine, in particular, whether any effect varies with the child's gender. We analyse data in regard to maternal smoking in pregnancy across two generations using the Avon Longitudinal Study of Parents and Childhood (ALSPAC) and consider fetal growth in regard to whether the study parents were exposed *in utero*, contingent on their own smoking habits in pregnancy.

## Methods

### Ethics

Ethical approval for the study was obtained from the ALSPAC Law and Ethics Committee and the Local Research Ethics Committees.

### Study sample

The data used in these analyses were collected as part of the Avon Longitudinal Study of Parents and Children (ALSPAC), which was designed to assess the ways in which the environment interacts with the genotype to influence health and development [8]. Pregnant women, resident in the study area in south-west England with an expected date of delivery between 1^st^ April 1991 and 31^st^ December 1992, were invited to take part. About 80% of the eligible population did so [9].

**Figure 1:**
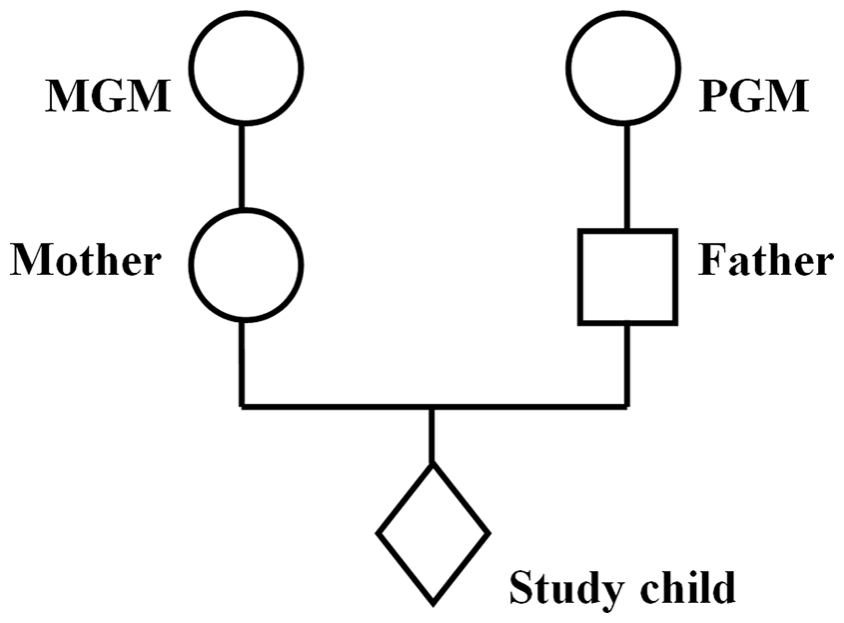
Diagram of intergenerational linkage.

Information collected from the parents during their study pregnancy included details of the maternal and paternal grandparents. Figure 1 illustrates the two pathways of possible influence of parental prenatal exposure to cigarette smoke on the study child that we will investigate in this paper, using the notation MGM and PGM to denote the maternal and paternal grandparents respectively.

### The exposures

The women and their partners were sent a number of questionnaires during pregnancy. These elicited information on their current smoking habits and those of their parents (i.e. the study grandparents). If they reported that their mothers had smoked, they were asked whether their mothers had smoked when expecting them – and, if so, were given the responses yes/no/don't know from which to select. Thus the parents who replied ‘don't know’, had a mother who smoked but the parent was unsure whether she had smoked during her pregnancy. We have analysed these data in two ways: (a) assuming that all these women did smoke during pregnancy, and (b) omitting the ‘don't knows’ from the analyses and only analysing those definitely reporting smoking status during the study pregnancy [this we have treated as a sensitivity analysis]. All mothers who themselves smoked during the study pregnancy were excluded from these analyses. Consequently we compared two groups of grandchildren: those whose grandmothers had smoked during the pregnancy resulting in their parent but whose mothers had not smoked with those whose parents had not smoked [MGM+M- with MGM–M- and PGM+M- with PGM–M-] respectively.

### Possible confounders

Other data used in the analyses include the study mother's parity (as ascertained from the maternal report of previous pregnancies resulting in either a live- or still-birth, and coded as 0; 1+); gestation (completed weeks: 39+; 37–38; ≤36); mother's partner smoking in pregnancy (primarily reported by partner, but maternal report was used if missing: yes; no); maternal age at the birth of the child (continuous); housing tenure as a measure of socioeconomic background (owned or mortgaged; rented public housing; all other), maternal education (highest level of educational attainment – in five levels of increasing achievement), maternal alcohol consumption when the mother first felt the baby move (not at all; <1 glass per week; and one or more glasses per week). Maternal birth weight was added as a separate confounder for a subsequent analysis.

### Outcome measures

At delivery the baby was weighed to the nearest gram; ALSPAC study staff visited the two main delivery hospitals each day and measured the crown-heel length and head circumference of available infants in a standardised manner [8]. Body mass index (BMI) was calculated as birthweight/length^2^ (g/m^2^).

In this study we have used Body Mass Index (BMI), rather than ponderal index (PI) as our measure of adiposity at birth for two reasons: (a) subsequent analyses will assess whether the association with birth BMI is reflected in childhood measures of adiposity, where BMI is more relevant; (b) although it is traditional to use PI at birth, there is little literature to justify this. It has been suggested that the criteria used to choose whether to use PI or BMI should be a measure that is independent of length [10]. We have assessed which of the two measures is independent of length at each gestation among ALSPAC births (Appendix) and found that BMI satisfies the independence requirement more closely than PI.

### Statistical analyses

Multivariable linear regression models assessed the grandchildren's adjusted mean birthweight, crown-heel length, head circumference and BMI by parental prenatal smoking exposure. All models were adjusted for maternal parity, maternal education, paternal smoking at the start of pregnancy and gestation with MGM-M- (or PGM-M-) as the reference categories. Additional models adjusted for maternal age, housing tenure and maternal alcohol use as well as maternal birthweight. Separate models were produced for each grandchild's sex and maternal parity. We tested any potential interaction between grandmaternal smoking and gender by entering interaction terms into a non-stratified (for gender) model.

## Results

Data on the smoking status of the maternal and paternal grandmothers when pregnant with the study mothers and fathers were available for 12707 and 9677 study children respectively (Table 1). Combining the groups where the grandmother was known to have smoked in pregnancy with those where she was known to have smoked but it was unknown whether or not in pregnancy identified 37.9% and 42.1% of maternal and paternal grandmothers as smoking prenatally.

**Table 1:** Grandmother's smoking in pregnancy leading to study parents' birth.

Smoking in pregnancy	Maternal grandmother	Paternal grandmother
No	62·1% (7888)	57·9% (5603)
Yes	23·4% (2968)	18·2% (1758)
Don't know^a^	14·5% (1851)	23·9% (2316)
All known	100% (12707)	100% (9677)

a Grandmother was a smoker but not known if she smoked in the relevant pregnancy.

The distributions of factors that are known to be associated with fetal growth are compared for those with and without a history of prenatal exposure of the mother [MGM+ and MGM–] and the father [PGM+ and PGM–] in Table 2. The study mothers who were exposed were more likely to have had previous births, lower education levels, live in public housing and drink slightly more alcohol. Their partners were more likely to smoke. However they were not less likely to deliver preterm. Their own birthweight showed a difference of 148 g between those whose mother did and those whose mother did not smoke, thus providing proof of validity of the MGM+ measure. For study fathers, there were similar associations with maternal parity, maternal education, housing tenure, and their own smoking habit. There was no association with their partner's alcohol consumption or her birthweight.

**Table 2:** The sample of the mothers who did not smoke in pregnancy according to the demographic variables related to each of the MGM and PGM histories.

	MGM+	MGM–	PGM+	PGM–
No. In Study	3034	6164	2860	4447
*Parity* a				
0	1224 (41.0)	2840 (46.7)	1255 (44.3)	2148 (48.7)
1+	1759 (59.0)	3242 (53.3)	1577 (55.7)	2261 (51.3)
		P<0.001		P<0.001
*Maternal education level* ^b^				
CSE or less	561 (20.0)	707 (12.2)	399 (14.8)	509 (11.2)
Vocational	282 (10.1)	476 (8.2)	281 (10.4)	323 (7.6)
O Level	971 (34.7)	2058 (35.4)	974 (36.1)	1447 (34.1)
A Level	637 (22.7)	1532 (26.4)	653 (24.2)	1132 (26.6)
Degree	350 (12.5)	1040 (17.9)	391 (14.5)	838 (19.7)
		P<0.001		P<0.001
*Gestation (weeks)* a				
39+	2317 (76.6)	4717 (76.8)	2180 (76.4)	3439 (77.5)
37/38	522 (17.3)	1043 (17.0)	501 (17.6)	716 (16.1)
<37	187 (6.2)	384 (6.3)	171 (6.0)	282 (6.4)
		P = 0.943		P = 0.254
*Partner smoking* a				
No	2052 (70.5)	4463 (75.0)	2072 (72.8)	3355 (75.8)
Yes	857 (29.5)	1489 (25.0)	766 (27.3)	1073 (24.2)
		P<0.001		P = 0.004
*Housing tenure* a				
Owned/mortgaged	2238 (76.4)	5093 (85.0)	2276 (81.3)	3749 (85.9)
Rented public	433 (14.8)	402 (6.7)	300 (10.7)	254 (5.8)
Rented private/other	260 (8.9)	496 (8.3)	224 (8.0)	360 (8.3)
		P<0.001		P<0.001
*Maternal alcohol* a				
Never	1436 (48.4)	3158 (52.5)	1379 (49.5)	1543 (35.5)
<1 glass per week	1084 (36.5)	2225 (51.2)	2068 (34.4)	1006 (36.1)
1+ glass per week	850 (15.2)	794 (13.2)	401 (14.4)	575 (13.2)
		P = 0.001		P = 0.244
*Maternal age* *(years)* ^b^	28.7 (4.9)	28.9 (4.5)	28.9 (4.7)	28.9 (4.5)
		P = 0.079		P = 0.502
*Maternal* *birthweight (Kg)* ^b^	3.172 (0.62)	3.32 (0.58)	3.26 (0.60)	3.28 (0.58)
		P<0.001		P = 0.314

a n(%); ^b^ mean (SD);

MGM maternal grandmother; PGM paternal grandmother; + smoked in pregnancy; − did not smoke in pregnancy.

### Maternal grandmothers

Compared to those with no data at all on MGM smoking, those with data were very similar (Table 3). However, the offspring were more likely to have parents who owned their home, to have better educated mothers, were less likely to have been born preterm, and had higher mean birthweights.

**Table 3:** Difference between study parents with and without details available of maternal grandmother's smoking history.

	History available	No history available
	N: 12707^a^	N: 2751^a^
*Features of pregnancy*		
Housing tenure	74·0% (9033/12215)	66·4% (857/1291)
(% mortgaged/owned)		
Mother drank alcohol	49·2% (6081/12374)	51·9% (298/574)
Study mother smoked in pregnancy	27·6% (3502/12701)	29·9% (443/1480)
Study partner smoked	38·1% (4599/12079)	37·1% (227/612)
Parity (% first child)	44·8% (5592/12487)	44·1% (283/642)
Maternal education > O level	35·8% (4169/11642)	28·6% (245/857)
Mean maternal age^c^	28·1±4·9 [12615]	27·0±5·3 [1460]
*Outcome of pregnancy*		
% male	51·7% (6526/14636)	50·0% (1117/2236)
% preterm ≤36wks^b^	6·2% (775/12583)	8·4% (124/1480)
Mean birth weight^c^	3390±577 [12455]	3307±605 [1445]
Mean birth crown-heel length^c^	50·6±2·5 [9593]	50·3±2·8 [984]
Mean birth head circumference^c^	34·8±1·6 [9736]	34·6±1·7 [1001]
Mean birth BMI^c^	13·3±1·5 [9492]	13·2±1·7 [967]

a Maximum n possible: Denominators for specific pregnancy and outcome variables vary due to missing data.

b Restricted to live-births, thus excludes miscarriages and late fetal deaths.

c mean ± SD [n].

Excluding information from pregnancies where the mother had smoked, the unadjusted mean birthweight of the boy infants was higher if the maternal grandmother had smoked during pregnancy (+86, [95% CI +49, +122]) g compared to those who did not smoke; this was only moderately attenuated upon adjustment (+61, [95% CI  = +30, +92]) g (Table 4). There was no pronounced indication of such a difference in birthweight of the girls (+14, [95% CI -15, +42]) g. Similarly there was an increase in the birth length (though not the head circumference) of the boys but not the girls. In spite of this increased birth length, there was an increase in mean BMI of the boys (adjusted +1.6; [95% CI +0.6, +2.6]) g/m^2^ but not of the girls [P for interaction  = 0.026]. These effects were robust to adjustment, and remained when the unknown status of pregnancy smoking of the grandmothers was omitted. Additional adjustment for maternal age, housing tenure and maternal alcohol use showed no difference in the results (data not shown). However further adjustment for maternal birthweight resulted in an increased effect in the birthweight of the boys from +61 g to +100 g [95% CI +61, +140] g; and for girls an increase from +14 g to +34 [95% CI −3, +70] g. A further analysis repeated that in Table 4, but restricted it to the infants whose fathers were non-smokers. The effect of MGM+ on birthweight remained in boys: after adjustment b  = +52 [95% CI +15, +89] g.

**Table 4:** Mean difference [95% CI] in birth measurements of children born to non-smoking mothers, comparing those where the child's grandmother had smoked with those who had not.

	MGM+ M- v. MGM– M-		PGM+ M- v. PGM– M-	
	Unadjusted	Adjusted^a^	Unadjusted	Adjusted^a^
**BIRTHWEIGHT (g)**				
Boy	**(4586)**	**(4125)**	(3633)	(3428)
	**+86**	**+61**	+38	+24
	**[+49, +122]**	**[+30, +92]**	[−2, +78]	[−9, +57]
Girl	(4442)	(3993)	(3546)	(3345)
	+9	+14	+18	+16
	[−24, +42]	[−15, +42]	[−18, +53]	[−13, +46]
**BIRTH LENGTH (cm x100)**				
Boy	**(3527)**	**(3209)**	(2810)	(2682)
	**+23**	**+19**	−2	0
	**[+5, +40]**	**[+2, +35]**	[−22, +18]	[−18, +17]
Girl	(3487)	(3183)	(2760)	(2629)
	+4	+4	+4	+11
	[−13, +21]	[−12, +20]	[−14, +22]	[−6, +27]
**HEAD CIRCUMFERENCE (cm x100)**				
Boy	(3580)	(3258)	(2856)	(2727)
	+8	+5	+6	+8
	[−3, +19]	[−5, +15]	[−7, +18]	[−3, +19]
Girl	(3537)	(3229)	(2799)	(2667)
	+2	+4	−1	+2
	[−8, +12]	[−5, +14]	[−12, +9]	[−8, +12]
**BMI g/m^2^**				
Boy	**(3491)**	**(3179)**	(2782)	(2656)
	**+2·1**	**+1·6**	+0·8	+1·0
	**[+1·1, +3·2]**	**[+0·6, +2·6]**	[−0·3, +2·0]	[−0·1, +2·0]
Girl	(3453)	(3153)	(2737)	(2606)
	+0.4	+0.5	+0.2	+0.3
	[−0·6, +1·5]	[−0·5, +1·5]	[−0·9, +1.3]	[−0·7, +1.3]

The number of individuals in each analysis are shown in round brackets.

a Adjusted for maternal parity, maternal education, partner smoked at start of pregnancy and gestation of study child.

[N.B. the data for birth length and head circumference are given in cm x 100 so as to aid interpretation].

### Paternal grandmothers

In contrast with the maternal grandmothers, the smoking status of paternal grandmothers did not appear to have any influence on the birth measurements of either sons or daughters (Table 4).

### Parity

The study of Misra et al [5] had indicated that the associations they found differed between the primiparous and multiparous study mothers. We therefore repeated the analyses separately for each of these parity groups (Tables S1–S2). Although the numbers involved were approximately halved in each group, there were similar associations for boys but not girls in mean birthweight, and mean BMI.

## Discussion

We have shown that when the mother does not smoke in pregnancy, there appears to be a discernible effect on her offspring of her own prenatal exposure. This was associated with an increased mean birthweight, birth length and BMI for her sons but not daughters, even after adjusting for factors such as maternal age, parity, socio-economic factors, and alcohol consumption. Similar associations were shown for children born to primiparae and those born to multiparae. The effect sizes for these associations were similar when sensitivity analyses restricted the information to those grandmothers who were definitely smoking during the mother's pregnancy (data not shown). In this study we did not control for maternal height, weight or BMI, nor for maternal birthweight as part of our primary analyses as we considered this likely to be an over-control. Nevertheless as part of a sensitivity analysis we repeated the analyses in Table 4 taking account of maternal height, weight and BMI: there was little overall difference in the results, in spite of reduced numbers; the boys continued to have increased mean birthweight (+50 [95% CI +18, +81]) g, birth length (+17 [95% CI +1, +34]) cm and BMI (+1.2 [95% CI +0.2, +2.3]) g/m^2^, whereas the girls did not: mean difference in birthweight (+19 [95% CI −11, +48]) g, birth length (+9 [95% CI −7, +25]) cm and BMI (+0.4 [95% CI −0.6, +1.4]) g/m^2^.

Our robust results on birthweight reflect those from three previous studies of maternal prenatal exposure to cigarette smoking, although none analysed their data separately for offspring sex [5]–[7]. One other MGM+M- smoking study has found an influence on a child outcome. It has been suggested that maternal smoking in pregnancy is associated with an increased risk of childhood asthma [11] but only one study [12] has analysed the risk of asthma distinguishing between the combinations as shown here. They found that, after allowing for race, gestation, and exposure to environmental tobacco smoke, compared with the MGM–M- group, the MGM+M- group were more likely to develop asthma (OR 1·8 [95% CI 1·0, 3·3]). Again no attempt was made to analyse the sexes separately.

### Differences in outcome by gender

It is not unusual for prenatal exposures to have differential sex effects. Maternal smoking has been associated with a greater growth reduction during fetal development in the male as compared with the female fetus [13]. There is also evidence that there is a gender specific increase in DNA methylation in cord blood at the IGF2 gene in response to maternal prenatal smoking, with boys being more affected than girls [14]. Maternal prenatal anxiety results in boys (but not girls) with higher symptoms of hyperactivity [15], and increased sympathetic nervous system response to a stress test [16]. However it is worth noting that some claims of sex-specificity of associations have failed to replicate [17]. In the present study the sex-specific outcome is in the *offspring* of the prenatally exposed individual; currently there are no comparable reports in humans. The nearest comes from the Överkalix historical studies, which show an association of the food supply during the prenatal development of the paternal grandmother with the mortality rate of her granddaughters but not grandsons, indicating that transmission is through the son [but not the daughter] of the woman prenatally exposed [2]. Of many rodent experiments showing differential outcomes by gender in response to parental exposure in utero, the most informative in the present context is a study in rats that shows that perinatal nicotine exposure induces asthma in second generation offspring [18]. In that study nicotine adversely affected lung function in both male and female offspring (F1) after prenatal exposure via the dam during pregnancy and until postnatal day 21. Interestingly the unexposed *offspring* of these F1 females (mated to their exposed F1 male siblings) had diminished lung function. In both the F1 and F2 offspring the males were more severely affected and tracheal constriction in response to acetylcholine was only seen in males, a sex-specific effect supported by increased tracheal fibronectin expression and other protein markers of tracheal responsiveness.

### Possible mechanisms of our findings

The findings in the literature [5]–[7] and in this study, of a change in birth measurements when their mothers were exposed *in utero*, raise the question as to possible mechanisms. Misra et al [5] regarded the increase in birthweight as a puzzle and wondered if the association was the result of residual confounding. The sex specific outcome in our study makes this explanation less likely and biological explanations for the transmission therefore need to be considered. There are three broad possibilities: (i) a cascade of metabolic knock-on effects involving initial somatic metabolic “programming” of the mother *in utero*; this would be expected to alter metabolic signals to her oocytes or across the placenta, thus influencing the growth of her own sons; (ii) a direct effect of grandmaternal smoking on oogenesis in the mother when she was a fetus; (iii) a combination of (i) and (ii).

In interpreting their finding of nicotine exposure producing comparable asthma outcomes in F1 and F2 generations in rats, Rehan et al [18] favoured direct epigenetic alterations of the germline, although only preliminary analysis of global DNA and histone methylation of the ovaries and testes was reported. Furthermore, their model of mating siblings (both of which had early life nicotine exposure) to produce the F2 generation complicates analysis of the route of any potential epigenetic transmission. Whilst pointing to biological transgenerational effects, this nicotine study is not comparable either to the present study or to others concerning the transgenerational effect of grandmaternal smoking in pregnancy [5], [6] in one important respect. In our study the MGM+M- combination is associated with an *increase* in birthweight and birth length of sons, even though maternal smoking produces a decrease in birthweight. This reversal in the outcome suggests an adaptive response down the generations, perhaps through mechanism (iii) above. Why such an adaptive response just affects sons is unclear. A recent review of sexual dimorphism emphasises [19] the differential epigenetic processes in the placenta, noting the abundance of X-linked genes involved in placentogenesis and the early unequal gene expression by the sex chromosomes between males and females.

It is worth noting that, in our study, the son's only X chromosome was exposed (during the mother's development), whilst daughters have both an exposed X from mother and a less/unexposed X from father. There is likely to be selection against sperm carrying the most chromosomal damage should the father be a smoker. Dosage compensation in females through X inactivation (whereby individual cells have either one or other X chromosome silenced epigenetically) might provide a mechanism for limiting the ‘adaptive’ increase in fetal growth in females.

Transgenerational adaptation to adverse exposures has been demonstrated in rodents, the most striking demonstrating increased protection against liver fibrosis down the (male line) generations [20]. Kuzawa has addressed this issue down the female line with respect to human fetal growth and nutrition [21]. He provides evidence suggesting that the flow of nutrients reaching the fetus provides an integrated signal of nutrition as experienced by recent matrilineal ancestors, which effectively limits the responsiveness to short-term ecologic fluctuations during any given pregnancy. However the human studies of ancestral famine on fetal growth produce complex results [22]. In essence, the Dutch famine of 1944–45 resulted in a tendency for the first born of women exposed *in utero* to be heavier than controls, whilst second and third born were lighter at birth. This context dependence is suggestive of adaptive responses.

### Strengths and weaknesses

The present study has the following strengths: (i) it is based on a large geographic population; (ii) information on parental and grandparental smoking was obtained prior to the birth of the study offspring, and therefore was not biased by the pregnancy outcome; (iii) birth measurements of the study child were obtained using standardised methods, which enabled accurate analyses of birth length and birth BMI; (iv) information on paternal prenatal exposures served as a comparison group. The transgenerational effect of maternal prenatal smoke exposure may have been socially patterned in some way, but if social patterning explained our findings one would expect a similar association with paternal exposure, yet this was not observed.

The study mothers were born between 1945 and 1975 and the fathers between 1902 and 1976. It should be remembered that smoking in pregnancy was first formally recognised as having possible adverse effects on fetal growth in 1957 [23], with public health advice first being provided in the USA by the Surgeon General in 1964 [24], but was not taken seriously by health providers in the UK until later. Indeed the rate of smoking in pregnancy in the two British national birth cohorts of 1958 and 1970 showed an increase in prevalence of smoking in 1970, and that those who smoked then did so more heavily [25]. It is likely that smoking women giving birth prior to 1970 rarely deliberately stopped smoking once they knew they were pregnant – therefore it is likely that the exposed study parents were exposed throughout pregnancy.

The disadvantage with this study is that although the data on prenatal smoking were collected from each of the study parents, no attempt was made at validation; however, the relationship between prenatal exposure and birthweight of the study parent and of the child showed predictive validity in regard to fetal growth (i.e. the birthweights of parents who were said to have smoked were lower than those who had said that they had not smoked – data not shown). We have not, as yet, been able to compare our sex- specific outcomes with those from another study although the overall results were similar to those of two other studies [5], [6] in regard to an overall increase in mean birthweight if the maternal grandmother but not the mother had smoked in pregnancy.

### Conclusion

We have shown that the non-smoking mother's own prenatal exposure to cigarette smoke is associated with an increased birthweight, and an increased birth BMI of the male offspring. This raises the question as to whether such an association continues throughout childhood and adolescence.

## Supporting Information

Table S1 **Mean difference [95% CI] in birth measurements of children born to non-smoking mothers, comparing those where the child's grandmother had smoked with those who had not when the child is the mother's firstborn.**
(DOCX)

Table S2 **Mean difference [95% CI] in birth measurements of children born to non-smoking mothers, comparing those where the child's grandmother had smoked with those who had not when the child is NOT the mother's firstborn.**
(DOCX)
