# Supplementary material for: Is the Growth of the Fetus of a Non-Smoking Mother Influenced by the Smoking of Either Grandmother while Pregnant?
Source: PLoS One. 2014 Feb 4;9(2):e86781. doi: 10.1371/journal.pone.0086781 (PMC3913581; doi:10.1371/journal.pone.0086781)
Supplement: Table S2 — Mean difference [95% CI] in birth measurements of children born to non-smoking mothers, comparing those where the child's grandmother had smoked with those who had not when the child is NOT the mother's firstborn. (DOCX) [file pone.0086781.s002.docx]

| **Table S2. Mean difference [95% CI] in birth measurements of children born to**  **non-smoking mothers, comparing those where the child’s grandmother had**  **smoked with those who had not when the child is NOT the mother’s firstborn** | | | | | |
| --- | --- | --- | --- | --- | --- |
|  | **MGM+ M- v. MGM- M-** | | | **PGM+ M- v. PGM- M-** | |
|  | **Unadjusted** | **Adjusted^a^** | | **Unadjusted** | **Adjusted^a^** |
| **BIRTHWEIGHT (g)** | | | | | |
|  |  |  |  | |  |
| Boy | +71 * | +55 * | +38 | | +24 |
|  | [+23, +118] | [+14, +95] | [-2, +78] | | [-9, +57] |
|  |  |  |  | |  |
| Girl | +21 | +20 | +18 | | +16 |
|  | [-23, +65] | [-20, +59] | [-18, +53] | | [-13, +46] |
|  |  |  |  | |  |
|  |  |  |  | |  |
| **BIRTH LENGTH (cm x100)** | | | | | |
|  |  |  |  | |  |
| Boy | +17 | +17 | -2 | | 0 |
|  | [-6, +41] | [-5, +38] | [-22, +18] | | [-18, +17] |
|  |  |  |  | |  |
| Girl | +3 | +4 | +4 | | +11 |
|  | [-21, +27] | [-19, +26] | [-14, +22] | | [-6, +27] |
|  |  |  |  | |  |
| **HEAD CIRCUMFERENCE (cm x100)** | | | | | |
|  |  |  |  | |  |
| Boy | +8 | +7 | +6 | | +8 |
|  | [-6, +23] | [-6, +20] | [-7, +18] | | [-3, +19] |
|  |  |  |  | |  |
| Girl | +6 | +8 | -1 | | +2 |
|  | [-8, +20] | [-5, +21] | [-12, +9] | | [-8, +12] |
|  |  |  |  | |  |
| **BMI g/m^2^** |  |  |  | |  |
|  |  |  |  | |  |
| Boy | +1.4 * | +1.4 * | +0.8 | | +1.0 |
|  | [+0.1, +2.8] | [+0.1, +2.7] | [-0.3, +2.0] | | [-0.1, +2.0] |
|  |  |  |  | |  |
| Girl | +0.8 | +0.7 | +0.2 | | +0.3 |
|  | [-0.8, +2.3] | [-0.7, +2.2] | [-0.9, +1.3] | | [-0.7, +1.3] |
|  |  |  |  | |  |
| *** statistically significant**  **^a^Adjusted for maternal education, partner smoked at start of pregnancy and gestation of study child** | | | | | |

[N.B. the data for birth length and head circumference are given in cm x 100 so as to aid viewing]
